# Jejunal atresia presenting with mesenteric cyst in a neonate: a case report

**DOI:** 10.1186/1757-1626-1-57

**Published:** 2008-07-23

**Authors:** A Pandey, AN Gangopadhyay, SP Sharma, VD Upadhyaya, V Kumar

**Affiliations:** 1 Department of Pediatric Surgery, Institute of Medical Sciences, Banaras Hindu University, Varanasi, 221005, U.P., India

## Abstract

Jejunoileal atresia is a congenital anomaly that is characterized clinically by bilious vomiting and abdominal distension. It has been associated with various congenital anomalies but its association with mesenteric cyst has only been reported sporadically. As this is a very rare entity, it is being reported with a brief review of literature.

## Introduction

Bilious vomiting is always of concern because of its association with surgical etiology. Smith considered bilious vomiting in conjunction with abdominal pain to be a surgical problem unless proved otherwise [1]. In neonatal period this vomiting is due to high bowel obstruction which is due to small bowel atresia, stenosis and malrotation [2]. We present a case of jejunal atresia (JA) that was associated with the mesenteric cyst, a very rare entity.

## Case presentation

A 12 hour old male neonate presented to the department with complaint of two episodes of bilious vomiting in last two hours. The patient was a full term normal vaginal delivery, delivered in the hospital. The family history was non contributing.

On examination, the general condition of the patient was good. His weight was 2.75 kg. There was no obvious congenital anomaly. On examination of the abdomen a cystic structure was felt in the right lumbar segment. X-Ray of the abdomen revealed three gas shadows in the left hypochondrium with no other gas shadow. A diagnosis of proximal small bowel atresia was made. No other investigation was done.

The patient was managed by nil by mouth and nasogastric tube placement. He was given intravenous (IV) fluid. The antibiotics given were ceftriaxone (75 mg/kg IV 12 hourly), amikacin(7 mg/kg IV 12 hourly) and metronidazole (7.5 mg/kg IV 12 hourly).

**Figure 1:**
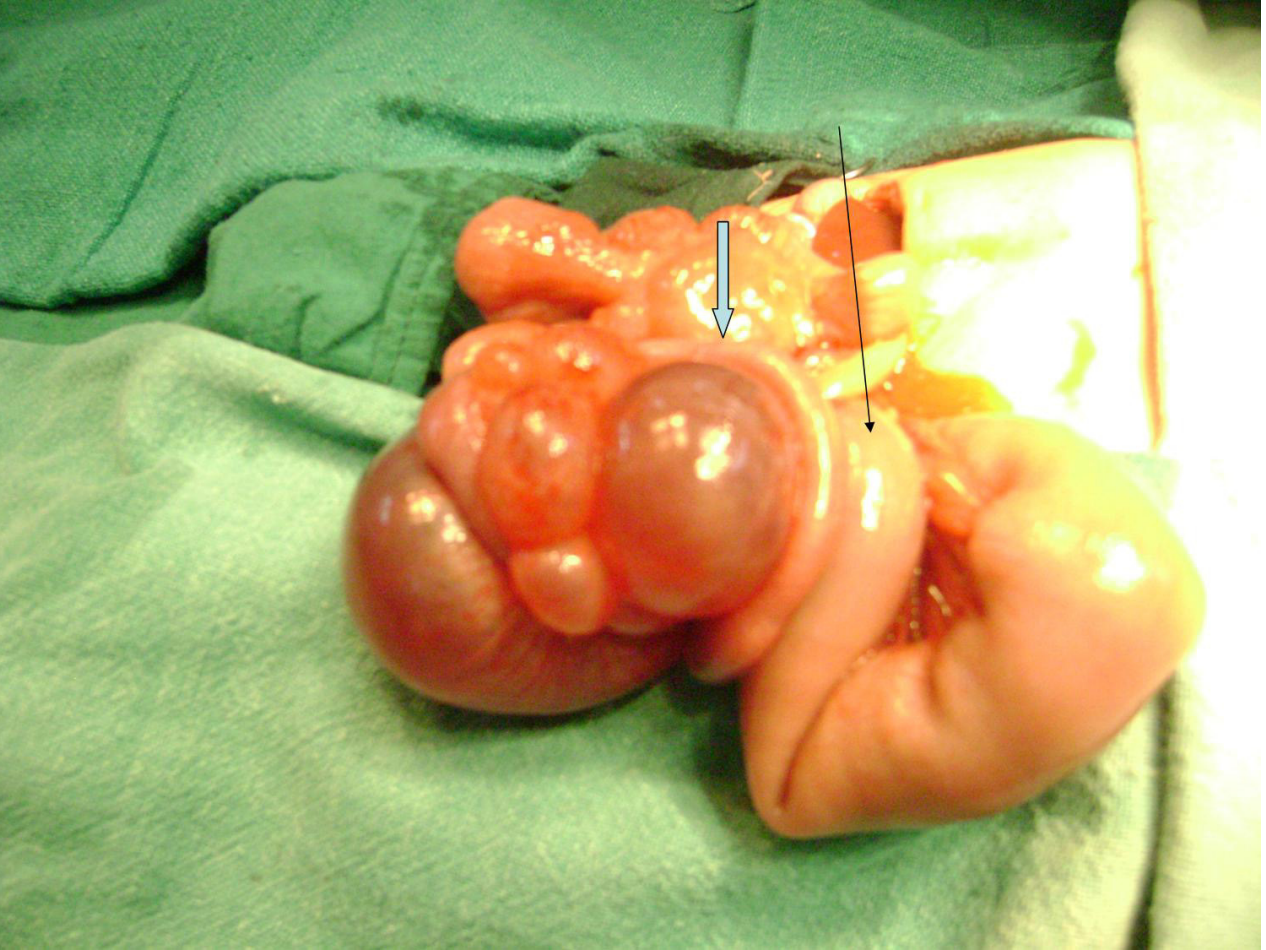
**Short title- Jejunal atresia with mesenteric cyst**. Peroperative photograph showing jejunal atresia with mesenteric cyst. The broad arrow is pointing towards the jejunal atresia and associated mesenteric cyst. The long and narrow arrow is pointing towards the proximal dilated jejunum.

The patient was operated after about 24 hours of presentation. Under general anesthesia, the right transverse supraumbilical incision was made. On exploration of the peritoneal cavity there was presence of a mesenteric cyst in the jejunal mesentery that was felt as the cystic structure on abdominal examination (figure 1). The jejunum associated with the mesenteric cyst was having a single type I atresia. The presence of atresia was 15 cm from the dudenojejunal flexure. The proximal bowel was dilated. The treatment included resection of the cyst along with the atretic bowel and proximal 7.5 cm of the dilated bowel and end to end single layer anastomosis by vicryl 5-0.

The post operative period was uneventful. The patient was allowed orally on 11^th ^post operative day. IV antibiotics were continued for 11 days followed by oral cefixime (4 mg/kg BD) for seven days. He was discharged on 14^th ^post operative day in satisfactory condition. The follow up of the patient is also satisfactory.

## Discussion

Jejunoileal atresia (JIA) is generally considered to result from intrauterine vascular disruptions to a segment of the developed intestine [3]. The incidence of JIA varies from 1:330 and 1:400 live births in some reports to between 1:1,500 and1:3,000 live births in others and JA is said to account for about 40–50% of these [4].

The clinical features of JA include bilious vomiting and upper abdominal distension. Our patient had bilious vomiting but no distension a he presented early in the course of the disease. Antenatally it may be associated with maternal polyhydramnios [5] but it was not present in our patient. On plain x-ray of the abdomen there is presence of few air fluid levels with no gas beyond that point [5]. The treatment is surgery. We resected the proximal 7.5 cm of dilated bowel so as to get rid of the possible complication of ineffective peristalsis of the grossly dilated segment [5].

JA has been associated with a number of other congenital malformations such as cystic fibrosis, malrotation, congenital heart disease, Down's syndrome, congenital dislocation of hips, anorectal and vertebral anomalies neural tube defect and microcephaly [4]. It has also been associated with biliary atresia, total colonic aganlionosis, intestinal neuronal dysplasia, polysplenia and bilateral athelia with choanal atresia [4-7]. The association of mesenteric cyst with the JA in the neonatal period is extremely rare with less than 10 cases reported in the literature [8,9].

Clinically most of the disruptive events observed at surgery in neonates with JIA are mechanical obstructions such as volvulus, herniation, constriction, and intussusception [3]. It is possible that the occurrence of the mesenteric cyst was the causative factor for the vascular compromise in our patient.

Thus to conclude our case is unique in the sense that it showed an association that is extremely rare and reinforces the mechanical obstruction theory for JA to occur.

## Consent

Written informed consent was obtained from the parents of the patient for publication of this case report and accompanying images. A copy of the written consent is available for review by the Editor-in-Chief of this journal.

## Competing interests

The authors declare that they have no competing interests.

## Authors' contributions

SPS, VDU and VK operated the patient and reviewed the literature. AP and ANG were major contributors in writing the manuscript. All authors read and approved the final manuscript.
